# PharmacoMicrobiomics or how bugs modulate drugs: an educational initiative to explore the effects of human microbiome on drugs

**DOI:** 10.1186/1471-2105-12-S7-A10

**Published:** 2011-08-05

**Authors:** Ramy K Aziz, Rama Saad, Mariam R Rizkallah

**Affiliations:** 1 Department of Microbiology and Immunology, Faculty of Pharmacy, Cairo University, 11562 Cairo, Egypt; 2 Department of Computer Science, San Diego State University, San Diego, CA, 92182, USA; 3 Department of Biology, School of Sciences and Engineering, The American University in Cairo, 11835 Cairo, Egypt; 4 Open Source Technologies Department, Information Technology Institute, 12577 Giza, Egypt

## Background

Pharmacogenomics investigates how variations within the human genome affect the action and disposition of drugs as well as drug tolerance [1]. Yet, variations within the human genome do not fully account for the tremendous phenotypic variations observed between individuals. Human-associated microbes, which exceed the human cells in number, significantly contribute to the effective human gene pool, and their combined genomes (known as the human microbiome) have not gained attention until recently. The Human Microbiome Project was launched in 2007 to catalogue the tremendous diversity of cultured and uncultured human-associated microbial communities residing in different human tissues, and to study the effect of microbial genes and genomes on human health and disease [2,3]. However, the effect of these microbes on drugs remains largely unexplored. Since microbes have complex metabolism, including an extraordinary ability to metabolize xenobiotics [4-6], they are expected to play a pivotal role in modulating the action, disposition, and toxicity of drugs with which they interact in different sub-ecosystems within the human body [7].

## Materials and methods

The PharmacoMicrobiomics initiative (http://pharmacomicrobiomics.org) is a research-based educational web platform that aims at exploring how microbes modulate drugs. The project was launched as an educational platform to introduce bioinformatics and microbial genomics to pharmacy students while benefiting the research community. The first step of this project was mining existing literature and extracting known microbe-drug interactions using a combination of keywords in an iterative process. The second step was the manual curation of the extracted literature data and their classification by drug classes, microbial families, and body systems (e.g., Table 1). The third step is the creation of a relational database that includes the microbes at different body sites and their effects on drugs’ pharmacokinetic and pharmacodynamic properties. Finally, participating students screen and attempt to isolate fecal microbes that alter a specific drug, and each student selects a drug class and a microbial species within a body site to examine their complex interaction *in vitro*.

**Table 1:** Examples of effects of gut microbes on drugs

CID	Drug	Body Site	Microbial effects	NCBI PMID
64982	Baicalin [Potential antioxidant, anti-inflammatory and liver tonic]	Gut	Gut microbes hydrolyze baicalin and enhance its absorption. Absence of gut microbiota resulted in lower levels of baicalin in plasma following oral administration [8].	11197087

2724385	Digoxin [Cardiac glycoside]	Gut	*Eubacterium lentum* is responsible for the difference in metabolite concentration of digoxin between North Americans and Southern Indians [9]	2759492

1794427	Chlorogenic acid [Antioxidant]	Gut	Variation in gut microbiome alters chlorogenic acid metabolism [10].	12771329

1983	Acetaminophen [Analgesic and antipyretic]	Gut	Acetaminophen toxicity is associated with elevated levels of p-cresol produced by some bacterial communities [4].	19667173

9064	(+)- catechin and (-)-epichatechins [Anti-oxidants]	Gut	In germ-free rats, (+)-catechins and (-)-epicatechins resulted in increase in the levels of liver CYP450 2C11, and (+) catechins caused elevation in the specific activity of liver UGT-Chloramphenicol [11].	12659723

5734	Zonisamide [Anticonvulsant]	Gut	Gut microbiota reduce zonisamide into 2-sulfomoyacetylphenol. Levels of 2-sulfomoyacetylphenol reportedly increased upon re-inoculation of germ-free rats with gut microbiota [12]	9231340

## Conclusion

The literature-mining steps of the pharmacomicrobiomics project have resulted in the initiation of a continuously updated web portal maintained by students (http://pharmacomicrobiomics.org/papers and http://pharmacomicrobiomics.com/examples.html). The project is expected to build a knowledge base that allows interested students and scholars, in the future, to predict the behavior of untested members of drug classes or unstudied microbial species, and to design laboratory experiments for testing these predictions.

**Figure 1:**
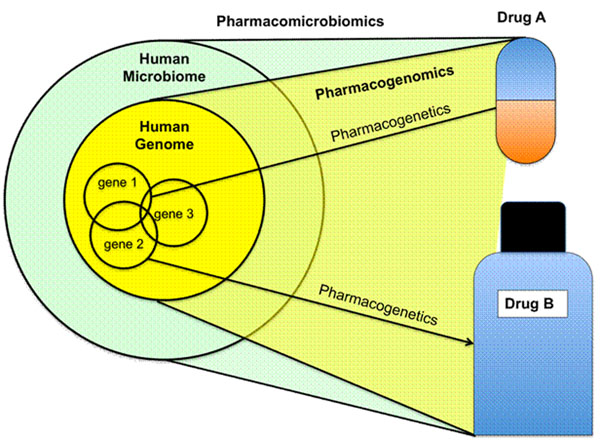
Pharmacogenetics investigates the effect of variations within single genes on drugs; pharmacogenomics investigates the effect of the sum of variations within the human genome on drugs; pharmacomicrobiomics investigates the effect of variations within the human microbiome on drugs.
